# Ethical Risks and Structural Implications of AI-Mediated Medical Interpreting

**DOI:** 10.2196/88651

**Published:** 2026-02-05

**Authors:** Alexandra Lopez Vera

**Affiliations:** 1 California University of Science and Medicine, 1501 Violet St, Colton, CA, 92324, United States, 1 9095809661

**Keywords:** artificial intelligence, AI-mediated interpreting, language access, health equity, clinical communication

## Abstract

Artificial intelligence (AI) is increasingly used to support medical interpreting and public health communication, yet current systems introduce serious risks to accuracy, confidentiality, and equity, particularly for speakers of low-resource languages. Automatic translation models often struggle with regional varieties, figurative language, culturally embedded meanings, and emotionally sensitive conversations about reproductive health or chronic disease, which can lead to clinically significant misunderstandings. These limitations threaten patient safety, informed consent, and trust in health systems when clinicians rely on AI as if it were a professional interpreter. At the same time, the large data sets required to train and maintain these systems create new concerns about surveillance, secondary use of linguistic data, and gaps in existing privacy protections. This viewpoint examines the ethical and structural implications of AI–mediated interpreting in clinical and public health settings, arguing that its routine use as a replacement for qualified interpreters would normalize a lower standard of care for people with Non-English Language Preference and reinforce existing health disparities. Instead, AI tools should be treated as optional, carefully evaluated supplements that operate under the supervision of trained clinicians and professional interpreters, within clear regulatory guardrails for transparency, accountability, and community oversight. The paper concludes that language access must remain grounded in human expertise, language rights, and structural commitments to equity, rather than in cost-saving promises of automated systems.

## Introduction

Artificial Intelligence (AI) is rapidly being integrated into public health practice [1]. Among its most visible and controversial uses are AI-mediated interpreting services, including real-time translation platforms and chatbot-based tools [2]. These technologies are promoted as scalable solutions to improve access for individuals with Non-English Language Preference (NELP), a population estimated to include more than 25 million people in the United States [3]. However, the use of these systems for medical interpretation raises immediate ethical concerns related to accuracy, autonomy, and equity. Acknowledging these realities, this viewpoint focuses not on whether AI tools can be preferable to no interpretation at all, but on the ethical and structural risks of normalizing AI-mediated interpreting as an acceptable substitute for qualified language services in routine clinical care.

In light of these concerns, uncritical adoption of AI interpreting poses ethical and structural risks, particularly for patient safety, autonomy, and equity [4]. Unlike professional interpreters who are trained to manage cultural nuance and medical terminology [5], AI systems rely on training data that often underrepresent Indigenous languages, regional dialects, and community-specific expressions [6]. Errors in translation can compromise informed consent, distort sensitive conversations about reproductive health or chronic disease, and undermine trust in both clinical encounters and public health communication [7].

These concerns are reflected in current evaluations of AI translation tools. Systematic reviews show that although AI translation tools can perform reasonably well when translating from English, accuracy declines substantially when translating into English, particularly for non-European languages [8]. Technical research has documented incremental improvements in grammatical recognition, such as tense translation in Chinese-English systems, but these advances remain limited to controlled corpora (ie, collections of text and speech data used to develop and evaluate machine translation models) and fail to capture the cultural and contextual dimensions essential to health care [9]. The integrity of AI translation research has also been questioned due to persistent concerns regarding evaluation practices, transparency, and reproducibility in AI-based language systems [10]. Such developments highlight not only technical shortcomings but also broader concerns about hype, oversight, and accountability.

Taken together, these issues reveal why AI translation cannot be treated as a substitute for professional interpretation in public health practice. Instead, its use must be guided by ethics, equity, and structural competency, ensuring that efficiency and cost-effectiveness do not come at the expense of accuracy, patient rights, and trust. This viewpoint analyzes the ethical risks of AI-mediated interpreting, outlines guardrails for responsible implementation, and considers policy implications for equitable integration.

## Technical and Linguistic Limitations of AI Interpretation

The technical performance of AI interpretation tools reveals both progress and persistent shortcomings [8]. Most systems are built on large-scale neural machine translation models that optimize statistical accuracy across widely spoken languages [11]. However, this optimization produces systematic blind spots: performance is strongest for languages with abundant training data and weakest for low-resource and Indigenous languages [12]. In this context, “low-resource languages” refers to languages for which limited digitized text, speech data, or annotated training materials are available for AI model development. Such disparities are not trivial—they map onto global and domestic inequities, leaving the very populations most dependent on language access at greater risk of miscommunication. Although AI translation systems may perform comparatively better for high-resource languages such as Spanish, any potential benefit is highly context-dependent and limited to low-risk scenarios where professional interpretation is unavailable; differential performance across languages raises serious equity and safety concerns.

For example, consider a routine outpatient encounter in which a patient with NELP describes intermittent chest tightness using an idiomatic expression that, when rendered literally by an AI translation system, is conveyed as “discomfort” rather than “pressure.” The clinician, relying on the translated output, may interpret the symptom as benign and defer further evaluation. A professional interpreter, by contrast, would be trained to clarify the patient’s meaning, recognize the potential clinical significance, and convey the urgency embedded in the original phrasing. In this scenario, the translation error is subtle rather than overt, yet it meaningfully alters clinical interpretation and risk assessment, illustrating how AI-mediated interpreting can introduce safety risks without obvious signals of failure.

Apart from language availability, AI models struggle with the communicative complexity of health encounters. Clinical communication frequently involves layered terminology, idioms, and pragmatic features such as hedging or expressions of uncertainty [13]. Because most AI translation systems are still trained on broad, nonmedical data, they often produce literal word-for-word renderings rather than contextually accurate translations [14]. In clinical and public health settings, this can shift the tone and meaning of communication—for example, turning cautious or conditional medical advice into statements that sound definitive, or softening urgent guidance into something that appears optional. Such distortions not only change the information being conveyed but also risk undermining patients’ understanding, informed decision-making, and trust in health professionals.

Context dependence is another unresolved challenge. While technical evaluations often report improvements in grammatical recognition or lexical choice, these gains are typically demonstrated in isolated sentence-level translations [15]. Real encounters involve extended dialogue, code-switching, and back-and-forth clarification—conditions under which current systems exhibit degradation in coherence and consistency [14]. For example, terminology may be translated differently within the same conversation, leading to patient confusion about diagnoses, treatment instructions, or medication use.

Finally, AI translation models are not designed to detect when they are likely to fail. Unlike human interpreters, who can request clarification or signal uncertainty, the AI outputs are delivered with apparent confidence regardless of underlying accuracy [16]. This “confidence illusion” increases the danger of undetected errors in high-stakes environments such as emergency care or consent discussions.

Taken together, these limitations demonstrate that the technical progress of AI interpreting remains insufficient to guarantee accuracy, consistency, and safety in public health and clinical practice.

## Data Security and Confidentiality Risks

Beyond issues of accuracy, AI-mediated interpreting also raises serious concerns regarding data security and patient confidentiality. Most commercially available translation and chatbot systems are hosted on external servers and require transmitting speech or text data across networks outside the clinical environment. This creates risks of unauthorized access, data storage without consent, or secondary uses of sensitive information such as marketing or algorithm training [17]. In public health practice, these risks are not hypothetical—leaked or improperly managed health data can expose entire communities to stigma, discrimination, or even legal jeopardy.

Such vulnerabilities directly conflict with existing privacy frameworks such as the Health Insurance Portability and Accountability Act (HIPAA) in the United States, which mandates strict safeguards around the handling of protected health information [18]. Unlike professional interpreters, who are trained to maintain confidentiality and bound by institutional or legal standards, AI systems have no inherent mechanism for accountability when breaches occur [19]. Furthermore, patients may be unaware that their personal health details are being routed through third-party systems, limiting their ability to provide meaningful informed consent. Table 1 summarizes key risks and ethical implications of AI-mediated interpretation in public health.

**Table 1.** Risks and ethical implications of AI-Mediated interpreting in clinical Encounters.

Domain	Key risks identified	Clinical implications
Linguistic accuracy	Literal rendering; inconsistent term mapping; unflagged uncertainty (“confidence illusion”)	Incorrect clinical interpretation; inappropriate triage/management; documentation errors
Equity in access	Performance gaps by language data availability; limited support for Indigenous/low-resource varieties	Unequal communication quality; differential risk of error; exacerbation of disparities
Patient safety and informed consent	Distorted hedging/urgency; loss of pragmatic meaning in sensitive topics	Compromised informed consent; delayed diagnosis/treatment; avoidable harm
Confidentiality and data security	Third-party processing/storage; unclear retention/secondary use; weak auditability	Unauthorized disclosure risk; reduced willingness to disclose; legal/compliance exposure
Ethical and structural implications	Substitution for qualified interpreters; normalization of a lower standard for NELP^a^ patients	Erosion of language rights; reduced trust in institutions; reinforcement of structural inequities

a NELP: Non-English Language Preference.

These data governance gaps highlight that the risks of AI interpretation are not only linguistic but structural. Without enforceable standards for data handling, encryption, and storage, reliance on AI tools for medical or public health communication could compromise patient trust and institutional integrity, with downstream effects on care-seeking and participation in public health programs.

This table summarizes key domains of risk associated with AI-mediated interpreting and their clinical implications. No numerical data were generated.

## Ethical Considerations

Ethics approval was not applicable as this viewpoint does not involve human participants, human data, human tissue, or any identifiable personal data.

## Conclusion

AI-mediated interpreting illustrates the tension between technological innovation and public health responsibility. These tools expand access and promise efficiency for populations with NELP, but their current limitations—ranging from linguistic inaccuracies to data security vulnerabilities—pose risks that threaten patient safety, confidentiality, and trust. Treating AI as a replacement for professional interpretation risks normalizing inequities and undermining ethical obligations to protect vulnerable communities.

The path forward is not outright rejection but cautious, principled integration. AI tools may serve as supplemental aids when professional interpreters are unavailable, but their deployment must be governed by enforceable standards for accuracy, transparency, and privacy. Some limited applications—such as translation of standardized materials or carefully constrained use in high-resource languages—may warrant cautious exploration. Even in these contexts, however, variability in dialect, health literacy, and clinical framing limits assumptions of safety and underscores the need for clear boundaries and oversight rather than broad endorsement.

Responsibility for establishing and enforcing these guardrails is shared. Health systems and public health agencies play a central role through procurement decisions, staff training, and oversight of clinical use, while technology vendors must ensure transparency around model limitations, data handling, and intended use. Regulators and accrediting bodies can reinforce these efforts by setting minimum standards for certification and independent auditing, particularly for tools used in high-stakes clinical and consent-related encounters. Framing AI-mediated interpreting as a patient safety issue, rather than solely a cost-saving tool, is essential to ethical and equitable implementation.

Recognizing language access as both a structural competency and a patient right is essential. Ultimately, aligning technological adoption with ethical safeguards and obligations will determine whether AI in public health functions as a bridge to equity or a source of new disparities.

## Abbreviations

AI: artificial intelligence
HIPAA: Health Insurance Portability and Accountability Act
NELP: Non-English Language Preference
